# Draft Genome Sequences of Pseudomonas fluorescens Strains SF39a and SF4c, Potential Plant Growth Promotion and Biocontrol Agents

**DOI:** 10.1128/genomeA.00219-15

**Published:** 2015-03-26

**Authors:** Lindsey K. Ly, Grace E. Underwood, Lucy M. McCully, Adam S. Bitzer, Agustina Godino, Vanni Bucci, Christopher J. Brigham, Analía Príncipe, Sonia E. Fischer, Mark W. Silby

**Affiliations:** a Department of Biology, University of Massachusetts Dartmouth, North Dartmouth, Massachusetts, USA; b Facultad de Ciencias Exactas, Físico-Químicas y Naturales, Universidad Nacional de Río Cuarto, Río Cuarto, Córdoba, Argentina; c Department of Bioengineering, University of Massachusetts Dartmouth, North Dartmouth, Massachusetts, USA

## Abstract

Pseudomonas fluorescens SF4c and SF39a, strains isolated from wheat rhizosphere, have potential applications in plant growth promotion and biocontrol of fungal diseases of crop plants. We report the draft genome sequences of SF4c and SF39a with estimated sizes of 6.5 Mb and 5.9 Mb, respectively.

## GENOME ANNOUNCEMENT

Pseudomonas spp. produce a variety of metabolites involved in biocontrol of plant diseases (1, 2). For example, Pseudomonas protegens (formerly Pseudomonas fluorescens) Pf-5 produces 2,4-diacetylphloroglucinol (2,4-DAPG), pyrrolnitrin, pyoluteorin, hydrogen cyanide, the siderophores pyochelin and pyoverdine, the cyclic lipopeptide orfamide, and rhizoxin derivatives (3–6).

P. fluorescens strains SF4c and 39a, isolated from the rhizosphere of wheat in Argentina (7), both promote wheat growth (7). SF4c also promotes tomato growth and inhibits plant-pathogenic *Rhizoctonia* solani and Sclerotinia minor (8), indicating biocontrol and plant growth-promoting potential. SF4c produces a bacteriocin similar to a phage-like pyocin of P. aeruginosa, which has antibacterial activity against some phytopathogenic pseudomonads, and is induced by DNA damage, suggesting involvement of the SOS response (9).

Genomic DNA of P. fluorescens SF39a and SF4c was extracted using Promega’s Wizard Genomic DNA purification kit. Libraries were generated using Illumina’s Nextera XT sequencing preparation kit and sequenced at the Tufts University Genomics Core using an Illumina MiSeq. For SF4c we obtained 3,382,840 2 × 250-bp reads (117-fold coverage), and for SF39a there were 3,439,664 2 × 250-bp reads (134-fold coverage). Genomes were assembled using CLC Genomics Workbench version 7.5. The SF4c assembly comprises 47 contigs (from 764 to 757,140 bp), and SF39a has 50 contigs (from 697 to 764,320 bp).

The draft genome sequence of SF4c comprises 6,507,013 bp (60.5% G+C content), while the SF39a sequence comprises 5,884,230 bp (60% G+C content). Both genomes were annotated using NCBI’s PGAP pipeline. For SF4c, 5,673 protein-coding sequences, 55 tRNA genes, and 16S, 23S, and 5S (two copies) rRNA genes were predicted. For SF39a, 5,045 protein-coding sequences, 54 tRNA genes, and 16S, 23S, and 5S (two copies) rRNA genes were predicted.

The *hcnABC* operon for hydrogen cyanide (10) was detected in SF4c (contig 7) and SF39a (contig 15), confirming previous work in SF4c (8). Although genes specifying pyrrolnitrin (11), phenazine (12), or 2,4-DAPG (13) have been characterized in related species, previous work indicated that SF4c does not have these genes (8). These results were confirmed, as was the absence of these genes in SF39a. Genes for pyoluteorin (14) are also absent from both strains.

In P. fluorescens Pf0-1, SBW25, and Q8r1-96, and P. protegens Pf-5, pyocin-like prophages are integrated between *mutS* and *recA*–*recX* (15). The genome sequence confirms that previously identified genes encoding a lytic system (*hol* and *lys* genes), the repressor gene (*prtR*), and a structural gene from R-type pyocin (9) are associated with a pyocin-like prophage integrated in the same locus (between QS95_21670 and QS95_21900 in contig 18). The presence of another phage-like bacteriocin (F-type pyocin) cluster was also identified in the same region of contig 18. Similar results were found in the strain SF39a (contig 17).

These strains expand the arsenal of bacteria, which may allow reduced fertilizer and chemical pesticide use. The genome sequences will enhance analysis of mechanisms of biocontrol and plant growth promotion, and deepen knowledge of P. fluorescens genomics.

### Nucleotide sequence accession numbers.

These whole-genome shotgun projects have been deposited at DDBJ/EMBL/GenBank under the accession numbers JTGG00000000 (SF39a) and JTGH00000000 (SF4c). The versions described in this paper are versions JTGG01000000 and JTGH01000000, respectively.
